# Management of selected waste generated during cable production

**DOI:** 10.1007/s11356-023-31448-x

**Published:** 2023-12-15

**Authors:** Waldemar Studziński, Alicja Gackowska, Michał Dadzibóg

**Affiliations:** 1 Faculty of Chemical Technology and Engineering, Bydgoszcz University of Science and Technology, Seminaryjna 3, 85-326 Bydgoszcz, Poland; 2 TELE-FONIKA Kable S.A., Bydgoszcz Plant, Fordońska 152, 85-197 Bydgoszcz, Poland

**Keywords:** Organic waste, Fractionation, Chromatographic analysis, Waste management, Circular economy, Acetophenone, Aluminum sludge

## Abstract

The subject of the research was the recovery of raw materials from waste generated in the production of cable insulation and the management of aluminum sludge. It was found that 49% (w/w) acetophenone, 6.8% (w/w) α-methylstyrene, and 17.2% (w/w) cumyl alcohol can be recovered from waste with a loss on ignition of 95% and used in various industries. A gas chromatograph equipped with a mass spectrometry detector was used to identify the recovered compounds. A waste distillation process was proposed to remove the water layer and obtain a concentrated acetophenone fraction. A method of neutralizing the water fraction and distillation residues is presented. The proposed waste management method is an alternative method to the currently used thermal transformation method. In turn, aluminum sludge was used to produce aluminum sulfate, which was used in the plant’s sewage treatment plant as a coagulant. The effect of this action was a reduction of 67% in the content of total iron, 60% of trivalent iron, and 32% of chemical oxygen demand. The above-mentioned examples of waste management are part of a closed-loop waste management strategy.

**Supplementary Information:**

The online version contains supplementary material available at 10.1007/s11356-023-31448-x.

## Introduction

Problems related to the management of industrial waste are currently one of the most important issues of modern chemical technology. The increase in the amount of generated waste related to global industrial development forces us to take decisive measures related to their reduction and rational use. In addition, public policy approaches based on the zero-waste concept aim to minimize the negative impact of waste generation and management on human health and the environment, as well as to reduce resource consumption (European Directive 2008/98/EC 2008). The transformation of waste to an environmentally safe form, as well as action aimed at reducing its amount, requires the use of many techniques and technologies. Due to the current model of waste management, which puts a strong emphasis on recovery, large-scale research is conducted on the search for methods of separation of substances with a high separation factor at the lowest possible cost, especially when valuable ingredients can be obtained from waste (Kesieme and Aral 2015; Quist-Jensen et al. 2016). The method of isolating useful compounds depends on their properties. Therefore, an important stage in the development of a waste management procedure is the analysis of its matrix. In addition, it is necessary to have knowledge about the conditions of waste generation; its physical, chemical, and biological properties; and the ecotoxicological risk and the possibility of transforming the waste into a less harmful form (Mymrin et al. 2018). The harmful effect of waste on the environment may result not only from its original composition but also from the presence of intermediate products that arise as a result of uncontrolled chemical reactions in the waste mixture.

**Fig. 1:**
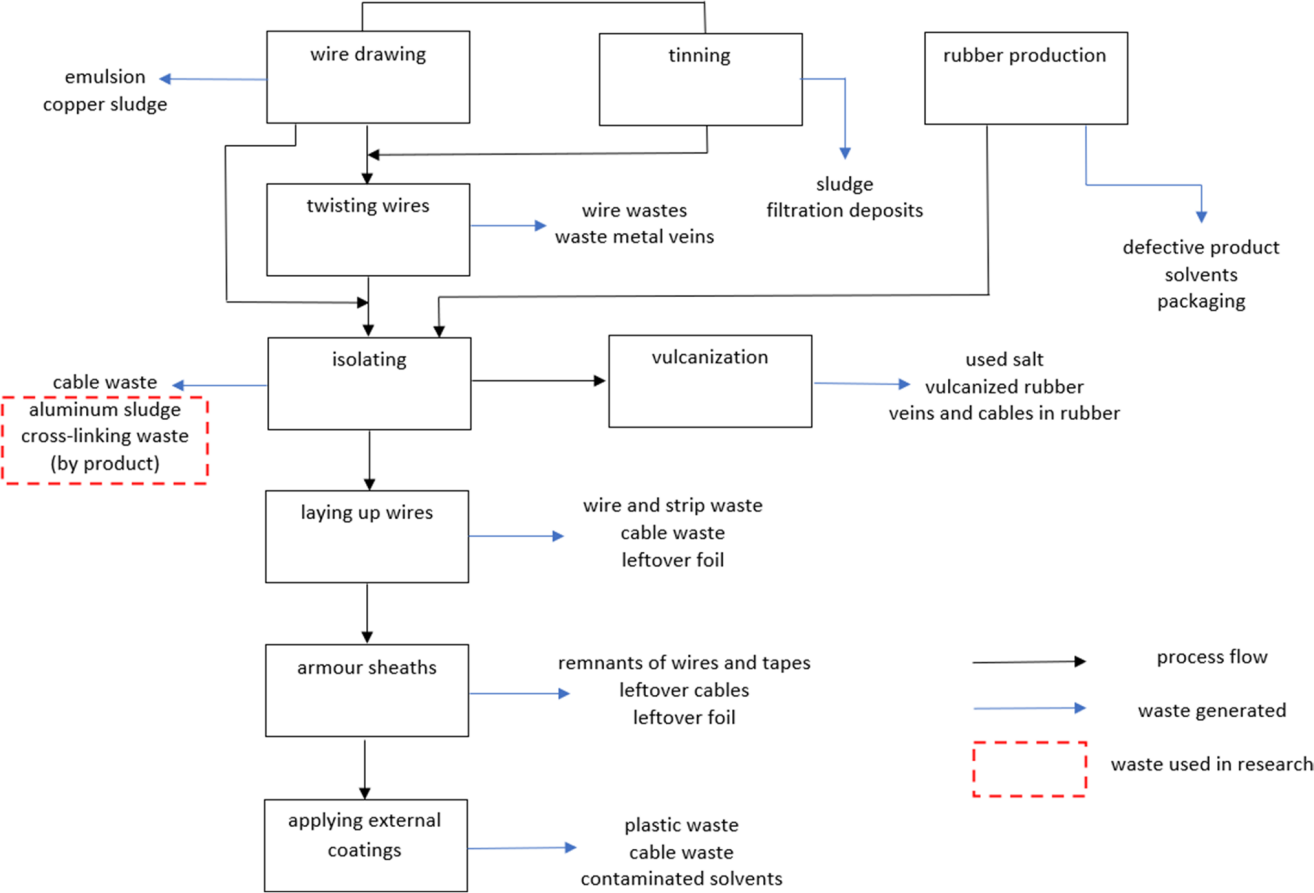
Block diagram of cable production

One of the industries where waste is generated is the cable industry. Cable production consists of a series of unit operations and processes. Each of these stages is associated with the formation of characteristic waste (Fig. 1). The waste that is generated is fragments of wires and cables, sludge related to the processing of wire, films and/or packaging materials, and by-products of insulation production.

**Fig. 2:**
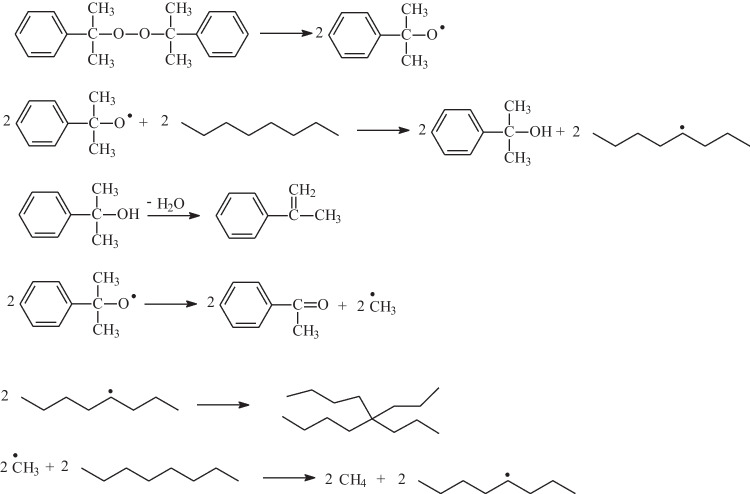
Diagram of cross-linking of polyethylene by the peroxide method (Andrews et al. 2006)

Waste wire strands and cables are recycled and managed (Xie et al. 2009; Liao et al. 2019; Trinh et al. 2021). The waste generated during the production of cable insulation is interesting. This waste is generated in the process of cross-linking polyethylene in the presence of dicumyl peroxide. This process takes place by means of radical reactions with the formation of a cumyl radical in the first stage, then a proton transfer from the polymer chain to the radical, and the formation of an active center on the polymer molecule. The last stage is the joining of polymer radicals (Fig. 2) (Andrews et al. 2006).

As a result of these transformations, in addition to the desired cross-linked polyethylene (XLPE), a by-product is produced (Online Resource 1). Currently, this waste is completely thermally neutralized. It should be emphasized that during the thermal neutralization of waste, valuable raw materials are often irretrievably lost which, had they not been lost, could have been reused after appropriate treatment. Therefore, the aim of the work is to check whether the by-product generated during the production of cable insulation can be managed in a different way than thermal destruction. Another interesting waste that can be used relatively easily is aluminum sludge (sodium aluminate). It is produced in the process of applying an aluminum sheath to the cable (Fig. 1; Online Resource 2).

The demand for aluminum in Europe is growing (Grimaud et al. 2018). The resources for aluminum mining are large, but limited. This metal is obtained from bauxite in the primary industry and from scrap in the secondary industry (Osoba et al. 2018). Since 99.7% pure aluminum is used for the production of cables, secondary aluminum does not meet the quality requirements. Thus, its recovery from sludge is economically unprofitable (Goodwin et al. 2005). The use of waste for the production of aluminum sulfate coagulant can bring measurable economic and ecological benefits. Aluminum salts are commonly used in the treatment of wastewater and raw water (Babatunde and Zhao 2007; Zhao et al. 2021). The use of sludge from cable production for the production of coagulants may, firstly, contribute to reducing the consumption of primary raw materials, and secondly, it may be a good method of waste management. The more so because, according to EU Directive 2008/98/EC, the method of landfilling is an undesirable form of waste management. Therefore, the next goal of the work was to check the possibility of using the sludge produced in the process of extrusion of aluminum coatings for the production of aluminum sulfate coagulant.

## Materials and methods

### Chemicals

Analytical standards α-methylstyrene (99%), acetophenone (99%), and cumyl alcohol (97%) were obtained from Sigma-Aldrich.

### Methodology

The following analyses of the by-product after cross-linking were performed: losses on ignition (Czylok FCF 5SHM muffle furnace), thermogravimetric analyses (Derivatograph Q 1500 D MOM, Budapest), chromatographic analyses (Hewlett-Packard type 6890 gas chromatograph equipped with a flame ionization detector (FID) and type 5890 equipped with mass spectrometry [MS] detector). The HP-1 column was used for the tests (25 m × 0.2 mm × 0.1 µm). Analyses were performed under the following conditions: sample volume 1 µl, injector temperature 250 °C, detector temperature 280 °C, oven temperature program 50–10 °C/1 min–250 °C. Helium was used as the carrier gas, flow of 1 ml/min. In the case of the identification of degradation products, the obtained MS spectra were compared with spectra from the NIST Mass Spectral Library 17.

In the next stage of the research, the authors prepared a coagulant based on aluminum sulfate contained in aluminum sludge. Water was added to 10 g of the sludge to 100 ml. The suspension was heated on a laboratory hotplate. In the next step, concentrated sulfuric acid was added until the suspension dissolved (the pH of the solution was below 4.0). The coagulant prepared in this way was used to purify actual sewage generated during cable production. The density of aluminum sludge was determined using the pycnometric method. Water content was determined gravimetrically. The aluminum content expressed as aluminum oxide was determined gravimetrically according to the procedure of precipitation of aluminum hydroxide and calcination of the precipitate to a constant mass. COD, total and trivalent iron, sulfates, and total suspended solids were determined in the sewage. COD was determined in accordance with the PN-ISO 15705:2005 standard. The USEPA SulfaVer 4 Method 8051 Powder Pillows procedure was used to determine sulfates. According to the laboratory’s instructions, total and trivalent iron were determined using the ammonium thiocyanate method. The NaOH content was determined by conductometric titration. Total suspended solids were determined per the guidelines in the PN-EN 872:2002 standard.

## Results

### Recovery of raw materials from by-product generated in the production of cable insulation

In order to achieve the assumed goal, it was necessary to conduct an initial waste analysis. This waste is an oily substance of a black and brown color. The preliminary chromatographic analysis and the losses after ignition (95%) indicate that organic compounds are the main components of the tested waste. Since the cross-linking process of polyethylene is carried out in the presence of dicumyl peroxide, it can be assumed that the waste is a mixture of organic compounds formed, inter alia, by the decomposition of peroxide. According to literature data, the products of peroxide decomposition can be α-methylstyrene and acetophenone (Di Somma et al. 2011; Krongauz et al. 2013; Conley et al. 2016; Valdes et al. 2016). Taking into account the above information and preliminary data on waste, a decision was made to carry out a distillation in order to check whether a fraction containing mainly dicumyl peroxide decomposition products can be separated from the waste. Therefore, the first distillation was carried out in three temperature ranges: up to 105 °C, from 105 to 250 °C, and above 250 °C. Six percent of a colorless fraction (up to 105 °C), 57% of a yellow fraction (105–250 °C), and 36% of still bottoms (< 250 °C) were obtained. The colorless fraction was mainly characterized by the content of the water layer (over 90%) and the colorless emulsion. As a result of the low volume and high degree of hydration, the colorless fraction can be treated by known chemical-biological methods together with other wastewater generated in the cable manufacturing plant. The distillation residue was brown in color, with a strong, unpleasant, slightly irritating smell, and consistency of tar, which was solid at room temperature. The analysis of this fraction showed that the losses after ignition are at the level of 99.5%. Similar results were obtained in the thermogravimetric analysis. It was shown that at the temperature of 815 °C, the sample was burned in 93%. The obtained results suggest that the bottoms can be co-incinerated, for example, in cement plants as an alternative fuel.

An interesting object of research was the yellow fraction (105–250 °C), which constituted a significant part of the waste. Chromatographic analysis of this fraction showed the presence of compounds with different boiling points. Therefore, the yellow fraction was subjected to another distillation in order to analyze its composition in detail. As a result of distillation, 11 fractions were obtained (Table 1).

**Table 1:** Fractions obtained by distillation of the yellow fraction

Fraction number	1	2	3	4	5	6	7	8	9	10	11
Temperature range (°C)	105–110	111–150	151–170	171–180	181–190	191–194	195–205	205–210	211–215	216–235	236–250

**Fig. 3:**
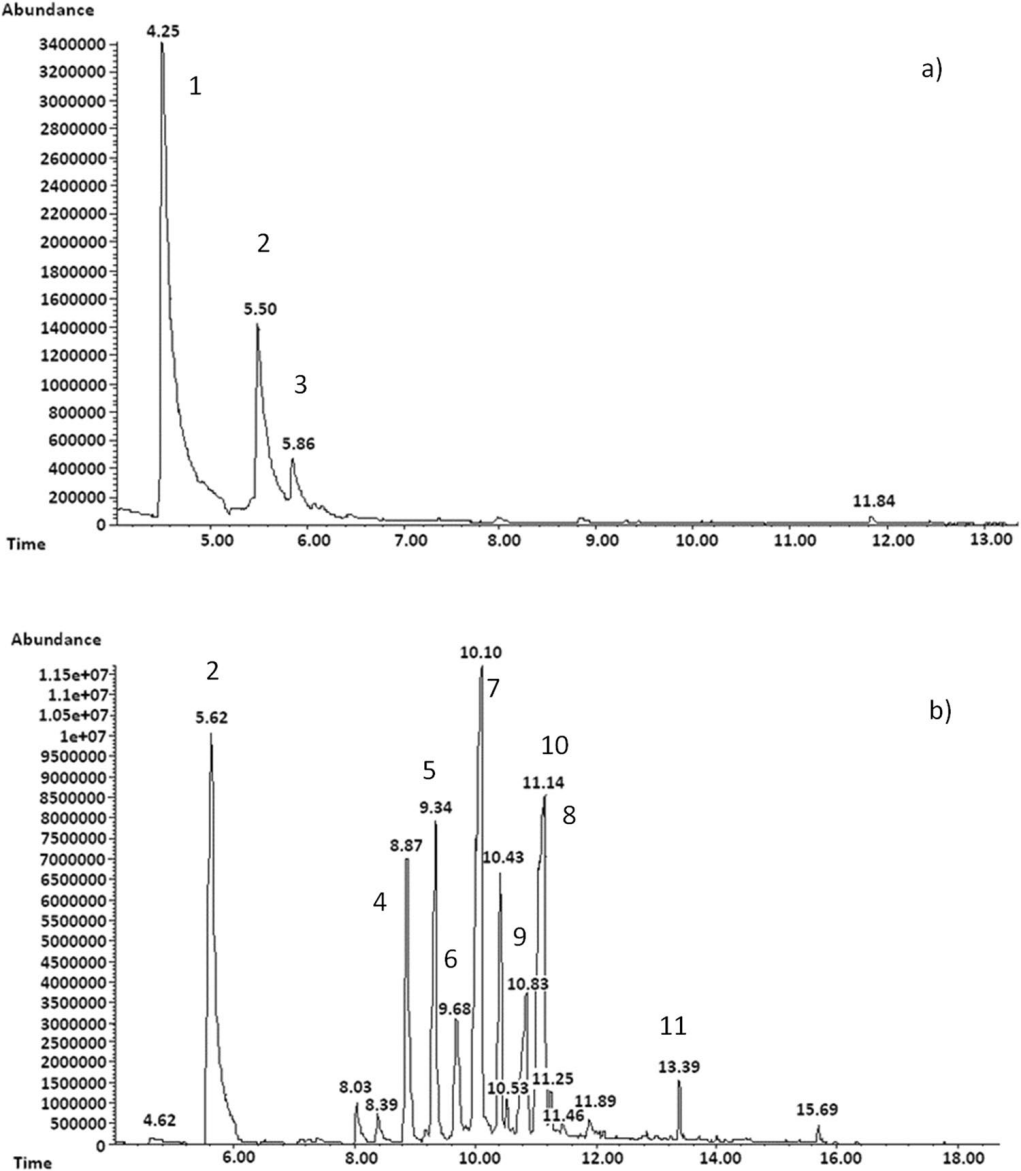
Chromatograms of fraction 5 (**a**) and fraction 11 (**b**). Identified compounds are the following: α-methylstyrene (1); acetophenone (2); cumyl alcohol (3); benzene, 1-(1-methylethenyl)-3-(1-methylethyl)- (4); benzene, 1-(1-methylethenyl)-4-(1-methylethyl)- (5); benzene, 1,3-bis (1-methylethenyl)- (6); ethanone, 1-(2,4,6-trimethylphenyl)- (7); ethanone, 1-[4-(1-methylethyl) phenyl]- (8); ethanone, 1-[4-(1-methylethyl) phenyl]- *t*_R_ = 9.4; ethanone, 1-[4-(1-methylethyl) phenyl]- (9); ethanone, 1,1′-(1,4-phenylene)bis- (10); quinoline, 3,4-dihydro-2,4,4-trimethyl- (11)

The obtained fractions were clear and differed in color. The first five solutions were colorless (105–190 °C), the next four were greenish (191–215 °C), and the last two were green-yellow (Online Resource 3). This proves that the chemical composition of the following distillates has changed. It was shown that the greatest amount of distillate was collected in the temperature range of 205–210 °C (fraction No. 8) and 181–190 °C (fraction No. 5) (Online Resource 4). In order to check the composition of the obtained fractions, chromatographic analyses were performed. For this purpose, a gas chromatograph with a flame ionization detector (FID) and a gas chromatograph coupled with a mass spectrometry (MS) detector were used. On the basis of the obtained results, it was found that the main components in the tested fractions are α-methylstyrene, acetophenone, and cumyl alcohol (Fig. 3a). In fraction 11, which was collected in the temperature range of 236–250 °C, the following compounds were also identified: benzene, 1-(1-methylethenyl)-3-(1-methylethyl)-; benzene, 1-(1-methylethenyl)-4-(1-methylethyl)-; benzene, 1,3-bis (1-methylethenyl)-; ethanone, 1-(2,4,6-trimethylphenyl)-; ethanone, 1-[4-(1-methylethyl) phenyl]-; ethanone, 1,1′-(1,4-phenylene)bis-; quinoline, 3,4-dihydro-2,4,4-trimethyl- (Fig. 3b). Information on the identified compounds and major *m/z* peaks is provided Online Resource 5.

**Fig. 4:**
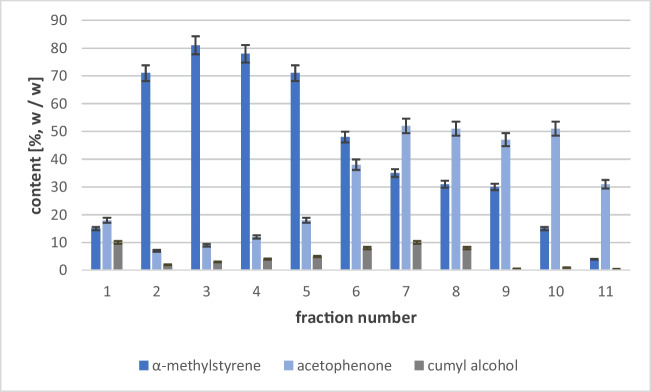
Percentage of acetophenone α-methylstyrene and cumyl alcohol in individual fractions

It has been shown that acetophenone is the dominant compound in the tested fractions. The highest concentration of acetophenone was recorded in fractions 6–10 (temperature range 191 to 235 °C), whereas the main component of fractions 3 and 4 was α-methylstyrene. Of the main components of the mixture, the lowest volume was observed for cumyl alcohol (mainly fractions 6, 7, and 8) (Fig. 4).

The conducted research shows that components that could be used in various industries can be recovered from the waste, which is now completely thermally neutralized. Acetophenone is used in the perfume industry as an additive to soap, creams, perfumes, and detergents and also used as a catalyst in the polymerization of olefins, in organic synthesis as a photo-oxidant (Soucy 2014; Kaur et al. 2019; Mohammadi Ziarani et al. 2020; Nagarajan et al. 2022). It is known that α-methylstyrene can be used as an additive to gasoline, or after hydrogenation to give cumene, which is used for the production of phenol (Mancuso et al. 2020). In turn, cumyl alcohol is used in the production of polymer compositions and in the production of specific polymers and resins (Akbarian et al. 2019).

**Fig. 5:**
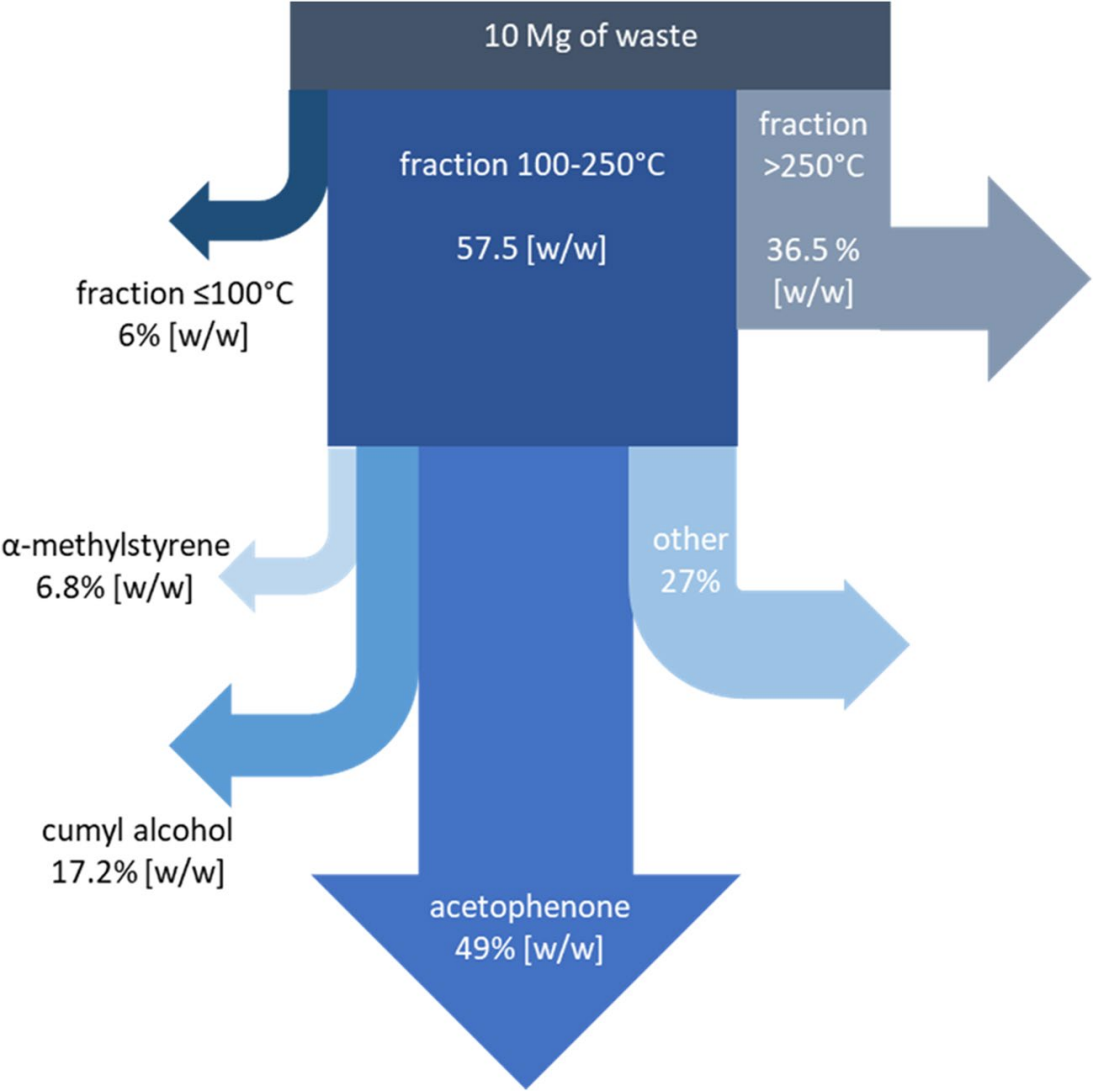
Recovery of acetophenone from waste generated in the process of producing cable insulation

During the production of cable insulation, approximately 10 Mg of waste is generated each year. The cost of thermal waste disposal is EUR 15,200. Distillation of waste into fractions ≥ 100 °C, 100–250 °C, and < 250 °C will result in obtaining a yellow fraction rich in acetophenone and reducing the amount of waste to 3.6 Mg, which translates into a reduction in the cost of disposal to EUR 5472. In turn, it was shown that the yellow fraction contains approximately 2817.5 kg of acetophenone, 391 kg of α-methylstyrene, and 989 kg of cumyl alcohol (Fig. 5). It was estimated that 400 kWh of energy was required to distill 10 Mg of waste, which translates into a cost of EUR 880. Isolation of acetophenone, cumyl alcohol, and α-methylstyrene from the yellow fraction can be performed using known methods, e.g., distillation under reduced pressure. Taking into account the fact that 1 kg of acetophenone costs EUR 46.9, 0.5 kg of cumyl alcohol costs EUR 970, and the cost of 1 kg of α-methylstyrene is EUR 92.07, the authors are convinced that the recovery of the substance will be economically profitable.

### The use of sodium aluminate recovered from the sludge generated in the production of cable sheaths

Realization of the goal required chemical analysis of waste, carrying out the reaction of obtaining aluminum sulfate from sludge (sodium aluminate) and checking its effectiveness in wastewater treatment. The influence of the coagulant on the wastewater treatment process was tested both on a laboratory scale and on a technical scale in the on-site wastewater treatment plant. The effectiveness of the coagulant was assessed on the basis of the degree of reduction of the following parameters in wastewater: total and trivalent iron, chemical oxygen demand, total suspended solids, and pH.

**Fig. 6:**
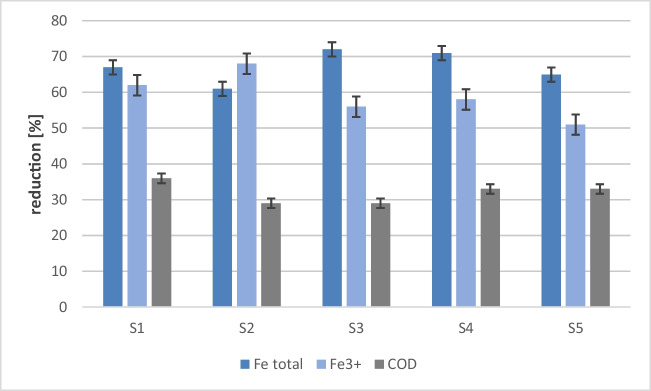
The effect of the addition of aluminum sulfate coagulant on the reduction of selected parameters in wastewater samples collected from five different points. Laboratory scale research

Chemical analysis of the sludge showed that it contains about 10% aluminum in terms of aluminum oxide, over 40% water, and about 8% NaOH (Online Resource 6) and is safe for use because toxic substances do not leach from it (Online Resource 7). The aluminum sludge was treated with sulfuric acid to obtain an aluminum sulfate solution, the effectiveness of which, in the first stage, was tested on a laboratory scale. For laboratory tests, wastewater collected from five points within the on-site wastewater treatment plant was used: S1, inlet to the biological wastewater treatment plant; S2, outlet from the container with a biological bed; S3, mixing chamber; S4, multi-stream settling tank inlet; S5, outlet from multi-stream settling tank (treated sewage) (Online Resource 8). The experiment was carried out in Imhoff funnels, into which 1 l of sewage and 1 ml of coagulant were introduced. Wastewater samples were collected at various intervals in the autumn and winter period. Total and trivalent iron, chemical oxygen demand, content of sulfate ions, and pH of sewage were determined in the tested samples (Online Resources 9–15). It was shown that the average reduction of total and trivalent iron was at the level of 67% and 59%, while the COD was at the level of 32% (Fig. 6).

Aluminum sulfate is an additional source of sulfate ions. There was an average increase in ion concentration of 36% (Online Resource 16), which did not exceed the limit value (500 mg/L) for the sewage treatment plant. The optimum pH of wastewater using aluminum sulfate coagulant is in the range of 6–9 (Kang et al. 2022). The pH has a great influence on the effectiveness of the removal of pollutants in the coagulation process. In a neutral and weakly alkaline environment, hydrolysis of Al^3+^ ions to Al (OH)_3_ takes place. Increasing the pH intensifies the hydrolysis of the coagulant, the neutral hydroxy complex of the Al(OH)_3_ cation is formed faster, and the force of which destabilizes the negative electrokinetic potential of the removed colloids is much lower than that of hydrolysis products with a positive electric charge (Nowacka et al. 2014; Pinotti and Zaritzky 2021). However, the condition for obtaining the appropriate amount of positive hydrolysis products is to reduce the pH value below 6. In an acidic environment, the effectiveness of the coagulation process in removing organic pollutants is influenced by favorable conditions for the formation of polymeric hydrolysis products of aluminum sulfate (VI) and a reduction in the degree of dissociation of organic substances (Kang et al. 2022). Therefore, reducing the pH value of treated wastewater increases the effectiveness of the neutralization of organic pollutants (Kurpińska 2020). In the case of humic iron compounds, the solubility of which decreases with increasing pH, the process of their adsorption on the surface of aluminum sulfate hydrolysis products plays an important role. It has been shown that the importance of the adsorption process is significant for pH above 6. However, it should be noted that at pH above 8, desorption of pollutants occurs as a result of the dissolution of aluminum hydroxide. The presented dependencies of the influence of pH on the coagulation process of pollutants confirm the obtained results of reduction of total Fe and Fe^3+^ at the level of 70% and COD at the level of 30% at the sewage pH of 7.6. By lowering or increasing the pH, we can cause the coagulant to transform into various forms which, under appropriate conditions, will hinder or accelerate the coagulation process (Yang et al. 2010). The addition of a coagulant slightly changed the pH of the tested sewage. Before the addition of the coagulant, the pH was 7.6, and after an hour, it had decreased on average by 0.3 (Online Resource 17).

**Fig. 7:**
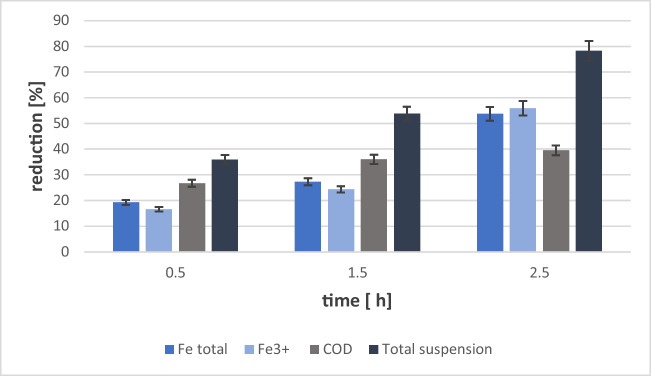
The effect of aluminum sulfate coagulant on the reduction of selected parameters in the wastewater. Research on a technical scale

The results obtained on a laboratory scale indicate that the adopted assumption of using waste as an additional source of coagulant may bring measurable benefits. Therefore, in the next stage, the obtained coagulant was used on a technical scale, three times; at 15-min intervals, aluminum sulfate in the amount of 5 l was introduced into the container (at the inlet to the treatment plant, sewage flow 3.75 m^3^ per hour). The results of the wastewater analysis before and after the addition of the coagulant are shown in Online Resources 18–20. An increase in the reduction of total and trivalent iron, COD, and total suspended solids was observed (Fig. 7).

After 2.5 h from the addition of the coagulant, the reduction of total iron was 53% and that of trivalent iron was 55%. The best results were obtained for the reduction of total suspended solids of 89%. On the other hand, the reduction of COD was at the level of 40%. The possibility of using non-commercial coagulants for wastewater treatment was also investigated by Babatunde et al. (2008) and Mora-León et al. (2022), who used sludge from water treatment as a raw material. Mora-León et al. (2022) obtained a similar reduction in total suspended solids as the authors, but they achieved much better results in the case of COD (90%). It can be assumed that the type of wastewater influences the reduction levels of chemically oxidizable pollutants. Mora-León et al. (2022) used municipal wastewater for research. However, in the case of the authors, it was sewage from cable production, which contains, inter alia, hard-to-decompose aromatic compounds. Therefore, the reduction of COD in the coagulation process may be difficult. This is confirmed by the results obtained by Jung et al. (2015), who reduced COD in textile wastewater using a non-commercial coagulant, achieving COD reductions of 60%. Finally, the authors compared the effectiveness of reducing contaminants on technical scale sewage using the tested and a commercial coagulant (Online Resource 21). In the study, it was found that the effectiveness of the coagulant obtained from sludge waste does not differ from that of commercial coagulant.

## Limitations and future perspectives

The work presents an alternative management of waste generated during cable production. The by-product generated during cross-linking of polyethylene has so far been thermally neutralized. The authors showed that using fractional distillation can reduce the overall waste mass and reduce disposal costs by 64%. As a result of distillation, a mixture rich in acetophenone, cumyl alcohol, and α-methylstyrene was obtained. The recovery and separation of valuable components require appropriate equipment and the development of techniques for purifying the separated fractions. Therefore, future research should focus on the separation and purification of acetophenone, cumyl alcohol, and α-methylstyrene. The next step is to re-analyze the costs and benefits of obtaining raw materials and compare the obtained results with the costs associated with thermal disposal. According to the information presented by the authors, it seems that the recovery of the three main components will be economically viable.

In turn, the use of sludge for the production of coagulants can, firstly, contribute to reducing the consumption of primary raw materials and, secondly, be a good way to manage waste. Another advantage is the fact that the method of obtaining the coagulant is relatively simple and can be used in the plant’s sewage treatment plant, which reduces the costs of waste transport and disposal. The advantage of this management method is that the use of aluminum sludge to produce a coagulant is not associated with high costs. It has been shown that the level of reduction in the content of total and trivalent iron, COD, and total suspension is comparable to the use of commercial coagulants.

The presented waste management methods are consistent with the circular economy strategy.

## Conclusion

The conducted research has shown that a fraction containing valuable raw materials, acetophenone, cumyl alcohol, and α-methylstyrene can be recovered from the by-product generated during the production of cable insulation. The remaining waste fractions can be managed in a relatively simple way. Moreover, it has been proposed to use an aluminum sludge to produce aluminum sulfate. The content of total and trivalent iron, COD, and total suspended solids can be reduced by means of a relatively simple coagulum. These ways of using waste fit into the circular economy strategy and, according to the authors, are economically profitable.

### Supplementary Information

Below is the link to the electronic supplementary material.

Supplementary file1 (DOCX 565 KB)

## Data Availability

The datasets used and/or analyzed during the current study are available from the corresponding author on reasonable request.
