# Supplementary material for: Management of selected waste generated during cable production
Source: Environ Sci Pollut Res Int. 2023 Dec 15;31(1):1664–73. doi: 10.1007/s11356-023-31448-x (PMC10789670; doi:10.1007/s11356-023-31448-x)
Supplement: Supplementary file 1 — Supplementary file1 (DOCX 565 KB) [file 11356_2023_31448_MOESM1_ESM.docx]

**Supplementary materials**


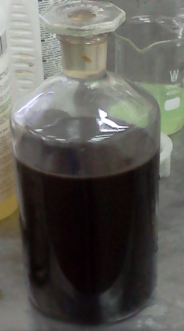


**Online Resource 1** By-product of the production of cable insulation


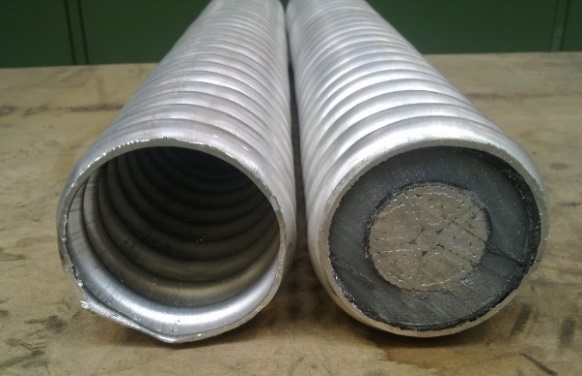


**Online Resource 2** Aluminum shell without a cable (left) and with a cable (right)


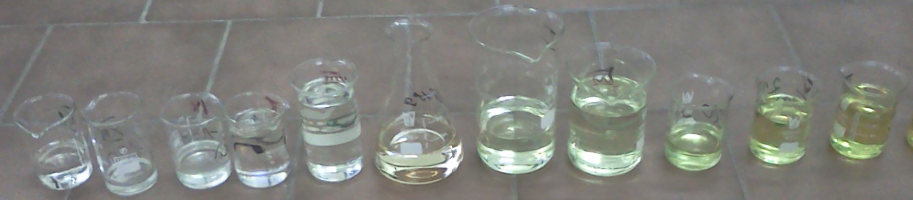


**Online Resource 3** Obtained fractions by distillation

**Online Resource 4** Graph of the volume of collected fractions

**Online Resource 5** List of Identified Compounds

| **Retention time T_R_** | **Compound** | **Summary formula** | **Structural formula** | **CAS** | **m/z** |
| --- | --- | --- | --- | --- | --- |
| 1 | α-Methylstyrene | C_9_H_10_ | 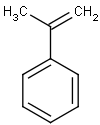 | 98-83-9 | 78,103,118 |
| 2 | Acetophenone | C_8_H_8_O | 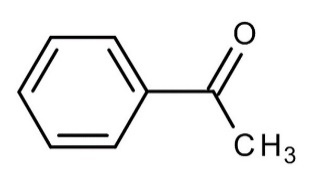 | 98-86-2 | 51,77,105,120 |
| 3 | Cumyl alcohol | C_9_H_12_O | 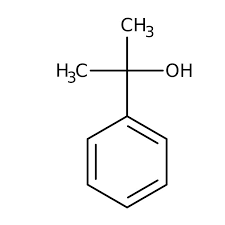 | 617-94-7 | 44,51,77,118,121 |
| 4 | Benzene,1-(1-methylethenyl)-3-(1-methylethyl)- | C_12_H_16_ | 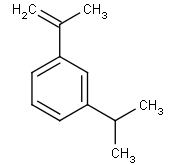 | 1129-29-9 | 117,145,160 |
| 5 | Benzene,1-(1-methylethenyl)-4-(1-methylethyl)- | C_12_H_16_ | 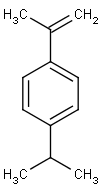 | 2388-14-9 | 105,145,160 |
| 6 | Benzene,1,3-bis(1-methylethenyl)- | C_12_H_14_ | 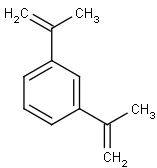 | 3748-13-8 | 128, 143, 158 |
| 7 | Ethanone,1-(2,4,6-trimethylphenyl) | C_11_H_14_O | 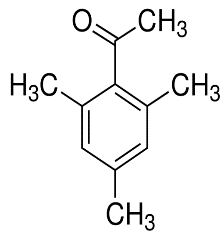 | 1667-01-2 | 43, 119, 147, 162 |
| 8 | Ethanone, 1-[4-(1-methylethyl) phenyl]- | C_11_H_14_O | 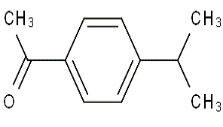 | 645-13-6 | 43, 91, 147, 162 |
| 9 | Ethanone, 1-[4-(1-methylethyl) phenyl]- | C_11_H_12_O | 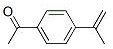 | 5359-04-6 | 115, 145, 160 |
| 10 | Ethanone, 1,1'-(1,4-phenylene) bis- | C_10_H_10_O_2_ | 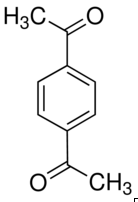 | 1009-61-6 | 43, 91, 147, 162 |
| 11 | Quinoline, 3,4-dihydro-2,4,4-trimethyl- | C_12_H_15_N | 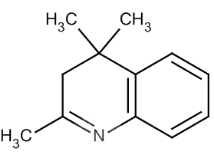 | 63177-93-5 | 35, 115, 158 |

**Online Resource 6** Results of chemical analysis of aluminum sludge

| **Sample number** | **Sample weight [g]** | **Density [g·ml^-1^]** | **Water content [%]** | **NaOH content [%]** | **Aluminum content as Al_2_O_3_ [%]** |
| --- | --- | --- | --- | --- | --- |
| 1 | 1.83 | 1.58 | 46.7 | 8.5 | 10.9 |
| 2 | 1.61 | 1.60 | 44.5 | 9.4 | 10.8 |
| 3 | 1.68 | 1.54 | 45.5 | 7.8 | 10.6 |
| 4 | 1.47 | 1.63 | 46.2 | 7.2 | 10.9 |
| 5 | 0.86 | 1.56 | 44.8 | 8.2 | 10.2 |
| 6 | 0.90 | 1.61 | 45.6 | 8.2 | 10.0 |
| Mean | 1.39 | 1.59 | 45.5 | 8.2 | 10.6 |

Selected physicochemical parameters of the water sample with the coagulant were analyzed. The aim of the research was to check whether the coagulant used was safe for the environment. 2 ml of coagulant was added to 1 liter of water. The system was stirred intensively. After 0.5 h, a sample was taken, selected physicochemical parameters were determined and the obtained results were compared with the guidelines for drinking water. The tests showed that the obtained values of the tested parameters were below the highest permissible concentration, except for total iron, the value of which was exceeded by 0.53 mg/l.

**Online Resource 7** Selected physicochemical parameters of the water sample with the coagulant

| **Physicochemical parameters** | **Tap water** | **Tap water with coagulant** | **Maximum permissible concentration** |
| --- | --- | --- | --- |
|  | **[mg/l]** | **[mg/l]** | **[mg/l]** |
| Chrome | 0.002 | 0.003 | 0.05 |
| Copper | 0.003 | 0.003 | 2.0 |
| **Additional physicochemical paramters** | **Tap water** | **Tap water with coagulant** | **Maximum permissible concentration** |
|  | **[mg/l]** | **[mg/l]** | **[mg/l]** |
| Manganese | 0.004 | 0.004 | 0.05 |
| Sulfates | 2.0 | 213 | 250 |
| Chlorides | 25.8 | 54.3 | 250 |
| Total iron | 0.05 | 0.73 | 0.2 |


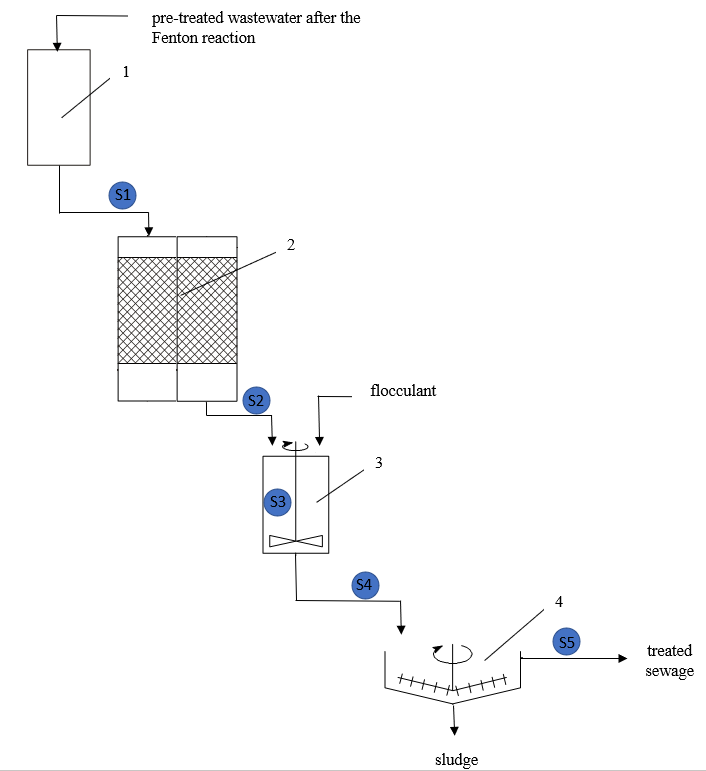


**Online Resource 8** Diagram of the on-site wastewater treatment plant with wastewater collection spots

**Marking of the places where sewage samples are taken for testing:**

**S1** – inlet to the biological wastewater treatment plant

**S2** – outlet from the container with a biological bed

**S3** – mixing chamber

**S4** – multi-stream settling tank inlet

**S5** – outlet from multi-stream settling tank (treated sewage)

**Test results on a laboratory scale**

*A. Results determination of total and trivalent iron*

**Online Resource 9** Total iron content without coagulant and after adding 1 ml of aluminum sulphate to 1l of sewage in the Imhoff funnel (experiment 1)

| **Wastewater sample collection point** | **Without any coagulant [mg·L^-1^]** | **One hour after adding the coagulant [mg·L^-1^]** | **Three hours after adding the coagulant [mg·L^-1^]** |
| --- | --- | --- | --- |
| S1 | 1.98 | 065 | 0..40 |
| S2 | 1.17 | 0.45 | 0.29 |
| S3 | 1.12 | 0.31 | 0.28 |
| S4 | 0.96 | 0.27 | 0.27 |
| S5 | 0.72 | 0.25 | 0.24 |

**Online Resource 10** Trivalent iron content without coagulant and after adding 1 ml of aluminum sulphate to 1l of sewage in the Imhoff funnel (experiment 2)

| **Wastewater sample collection point** | **Without any coagulant [mg·L^-1^]** | **One hour after adding the coagulant [mg·L^-1^]** | **Three hours after adding the coagulant [mg·L^-1^]** |
| --- | --- | --- | --- |
| S1 | 0.64 | 0.24 | 0.21 |
| S2 | 0.57 | 0.18 | 0.17 |
| S3 | 0.39 | 0.17 | 0.16 |
| S4 | 0.34 | 0.14 | 0.14 |
| S5 | 0.27 | 0.13 | 0.13 |

**Online Resource 11** Total iron content without coagulant and after adding 1 ml of aluminum sulphate to 1l of sewage in the Imhoff funnel (experiment 3)

| **Wastewater sample collection point** | **Without any coagulant [mg·L^-1^]** | **One hour after adding the coagulant [mg·L^-1^]** |
| --- | --- | --- |
| S1 | 1.10 | 0.46 |
| S3 | 0.90 | 0.40 |
| S5 | 0.78 | 0.28 |

**Online Resource 12** Total iron content without coagulant and after adding 1 ml of aluminum sulphate to 1l of sewage in the Imhoff funnel (experiment 4)

| **Wastewater sample collection point** | **Without any coagulant [mg·L^-1^]** | **One hour after adding the coagulant [mg·L^-1^]** |
| --- | --- | --- |
| S1 | 1.24 | 0.65 |
| S3 | 0.92 | 0.49 |
| S5 | 0.79 | 0.44 |

*B. Results Determination of Chemical Oxygen Demand (COD)*

**Online Resource 13** Chemical oxygen demand without a coagulant and after adding 1 ml of aluminum sulphate to 1l of wastewater in the Imhoff funnel (experiment 5)

| **Wastewater sample collection point** | **COD concentration in wastewater without coagulant [mg O_2_·L^-1^]** | **COD concentration in the wastewater one hour after adding the coagulant**  **[mg O_2_·L^-1^]** |
| --- | --- | --- |
| S1 | 216 | 137 |
| S2 | 149 | 106 |
| S3 | 153 | 109 |
| S4 | 155 | 104 |
| S5 | 146 | 97 |

*C. Sulphate determination results*

**Online Resource 14** The content of sulphates in sewage without coagulant and after adding 1 ml of aluminum sulphate to 1l of sewage in the Imhoff funnel (experiment 6)

| **Wastewater sample collection point** | **Sulphate concentration in the waste water without coagulant [mg·L^-1^]** | **Concentration of sulphates in the wastewater one hour after adding the coagulant**  **[mg·L^-1^]** |
| --- | --- | --- |
| S1 | 86 | 122 |
| S3 | 83 | 112 |
| S5 | 81 | 103 |

*D. Wastewater pH Results*

**Online Resource 15** Reaction of sewage without coagulant and after adding 1 ml of aluminum sulphate to 1l of sewage in the Imhoff funnel (experiment 7)

| **Wastewater sample collection point** | **Wastewater pH without a coagulant** | **The pH of the wastewater one hour after adding the coagulant** |
| --- | --- | --- |
| S1 | 7.65 | 7.22 |
| S3 | 7.67 | 7.30 |
| S5 | 7.61 | 7.42 |

## Online Resource 16 Percentage increase in the concentration of sulfate ions

**Online Resource 17** The influence of the coagulant on the change of the pH of sewage

**Test results on a technical scale**

1. *Results determination of total and trivalent iron*

At 10:45, 11:00, 11:15 (after the total iron content in the inlet sewage was above 3.0 [mg / l]), 5L of sulphate coagulant was added to the container with pre-treated sewage.

**Online Resource 18** Total and trivalent iron content in treated sewage (outlet from multi-stream settling tank) before adding the coagulant and after adding aluminum sulphate in the sewage treatment plant (experiment 8)

| **Wastewater sampling time** | **Without the addition of a coagulant** | | **After adding the coagulant** | |
| --- | --- | --- | --- | --- |
|  | **Total iron [mg·L^-1^]** | **Trivalent iron [mg·L^-1^]** | **Total iron [mg·L^-1^]** | **Trivalent iron [mg·L^-1^]** |
| 8:30 | 2.68 | 1.58 | - |  |
| 9:30 | 2.95 | 1.77 | - | - |
| 10:30 | 3.48 | 1.93 | - | - |
| 11:45 | - | - | 2.81 | 1.61 |
| 12:45 | - | - | 2.53 | 1.46 |
| 13:45 | - | - | 1.61 | 0.85 |

1. *Results Determination of Chemical Oxygen Demand (COD)*

At 11:00, 11:15, 11:30, 5L of sulphate coagulant was added to the container with pre-treated sewage.

**Online Resource 19** Chemical oxygen demand in treated sewage (outlet from a multi-stream settling tank) before adding a coagulant and after adding aluminum sulphate in a sewage treatment plant (experiment 9)

| **Wastewater sampling time** | **COD concentration in sewage [mg O_2_/l]** | |
| --- | --- | --- |
|  | **Without the addition of a coagulant** | **After adding the coagulant** |
| 10:45 | 86 | - |
| 12:00 | - | 63 |
| 13:00 | - | 55 |
| 14:00 | - | 52 |

1. *Results determination of total suspended solids*

At 11:00, 11:15, 11:30, 5L of sulphate coagulant was added to the container with pre-treated sewage.

**Online Resource 20** Total suspended solids content in treated sewage (outlet from multi-stream settling tank) before adding the coagulant and after adding aluminum sulphate in the sewage treatment plant (experiment 10)

| **Wastewater sampling time** | **Total suspended solids in sewage [mg·L^-1^]** | |
| --- | --- | --- |
|  | **Without the addition of a coagulant** | **After adding the coagulant** |
| 10:45 | 39 | - |
| 12:00 | - | 25 |
| 13:00 | - | 18 |
| 14:00 | - | 8.5 |

**Online Resource 21** Comparison of the tested coagulant with a commercial coagulant

|  | **Tested coagulant** | | | | **Commercial coagulant** | | | |
| --- | --- | --- | --- | --- | --- | --- | --- | --- |
| **Time [h]** | **Fe total** | **Fe^3+^** | **COD** | **Total Suspension** | **Fe total** | **Fe^3+^** | **COD** | **Total Suspension** |
| 0.5 | 19.2 | 16.6 | 26.7 | 35.9 | 17.7 | 15.6 | 26.4 | 37.1 |
| 1.5 | 27.3 | 24.3 | 36.0 | 53.8 | 29.4 | 25.9 | 23.5 | 47.6 |
| 2.5 | 53.7 | 55.9 | 39.5 | 78.2 | 48.8 | 46.5 | 41.4 | 70.9 |
